# Oridonin Inhibits *Mycobacterium marinum* Infection-Induced Oxidative Stress In Vitro and In Vivo

**DOI:** 10.3390/pathogens12060799

**Published:** 2023-06-03

**Authors:** Guangxin Chen, Ziyue Yang, Da Wen, Ping Li, Qiuhong Xiong, Changxin Wu

**Affiliations:** 1 Institutes of Biomedical Sciences, Shanxi University, Taiyuan 030006, China; 2 Shanxi Provincial Key Laboratory of Medical Molecular Cell Biology, Taiyuan 030006, China; 3 Shanxi Provincial Key Laboratory for Prevention and Treatment of Major Infectious Diseases, Taiyuan 030006, China

**Keywords:** *Mycobacterium marinum*, zebrafish, Ori, oxidative stress

## Abstract

Prior to the COVID-19 pandemic, tuberculosis (TB) was the leading cause of death globally attributable to a single infectious agent, ranking higher than HIV/AIDS. Consequently, TB remains an urgent public health crisis worldwide. Oridonin (7a,20-Epoxy-1a,6b,7,14-tetrahydroxy-Kaur-16-en-15-one Isodonol, C_20_H_28_O_6_, Ori), derived from the *Rabdosia Rrubescens* plant, is a natural compound that exhibits antioxidant, anti-inflammatory, and antibacterial properties. Our objective was to investigate whether Ori’s antioxidant and antibacterial effects could be effective against the infection *Mycobacterium marinum* (*Mm*)-infected cells and zebrafish. We observed that Ori treatment significantly impeded *Mm* infection in lung epithelial cells, while also suppressing inflammatory response and oxidative stress in *Mm*-infected macrophages. Further investigation revealed that Ori supplementation inhibited the proliferation of *Mm* in zebrafish, as well as reducing oxidative stress levels in infected zebrafish. Additionally, Ori promoted the expression of NRF2/HO-1/NQO-1 and activated the AKT/AMPK-α1/GSK-3β signaling pathway, which are both associated with anti-inflammatory and antioxidant effects. In summary, our results demonstrate that Ori exerts inhibitory effects on *Mm* infection and proliferation in cells and zebrafish, respectively. Additionally, Ori regulates oxidative stress by modulating the NRF2/HO-1/NQO-1 and AKT/AMPK-α1/GSK-3β signaling pathways.

## 1. Introduction

In 2021, an estimated 10.6 million individuals were afflicted with TB globally and 1.6 million people died from TB, while the incidence rate of TB increased by 3.6% from 2020 and 2021, reversing a decades long trend of approximately 2% annual decline [1]. Undeniably, TB remains a global public concern that poses a threat to human well-being [2,3]. Approximately 25% of the global population had been infected with the *Mycobacterium tuberculosis* (*MTB*) [4]; however, only a minority of those infected will develop to active tuberculosis (TB), while some individuals will spontaneously clear the infection [5,6]. Conventional TB treatments involve a regimen of multiple antibiotics, including isoniazid (INH), rifampicin (RIF), pyrazinamide (PZA), and ethambutol (ETH), and the therapeutic process lasts for 6 months [7], However, the treatment of TB caused by multidrug-resistant *MTB* and extensively drug-resistant *MTB* requires a longer therapeutic duration [8]. The lengthy treatment cycles and adverse side effects contribute to poor patient compliance rates, which, in turn, promote the emergence of multidrug-resistant TB (MDR-TB) [9,10]. The high rate at which mycobacterium tuberculosis acquires drug resistance necessitates a constant search for new anti-TB alternative drugs.

**Figure 1:**
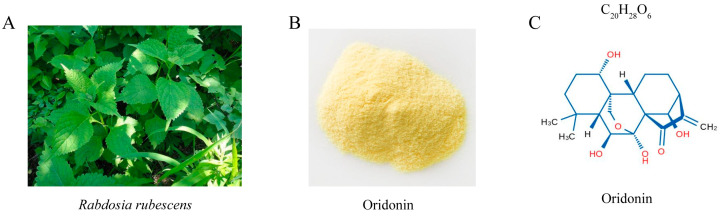
(**A**) *Rabdosia rubescens*, also named Donglingcao, produced inthe Taihang Mountains, Henan Province (Jiyuan, Linzhou, and Hebi city). (**B**) Ori, a light yellow acicular crystal. (**C**) The chemical structure of Ori (7a,20-Epoxy-1a,6b,7,14-tetrahydroxy-Kaur-16-en-15-one Isodonol, C_20_H_28_O_6_).

Ori, a bioactive ent-kaurane diterpenoid compound (Figure 1B,C), is the primary active constituent of *Rabdosia rubescens* (also named dong ling cao) leaves (Figure 1A), which have been widely used in traditional Chinese medicine [11,12]. There is mounting evidence that Ori exhibits potent antitumor activity [13,14], and inhibits the proliferation of over 20 human cancer cell lines, including lung cancer, prostate cancer, and colorectal cancer [15]. For centuries in China, Ori has been used clinically to treat inflammation-related diseases and remains one of the most popular herbs [16]. Ori exhibits a range of beneficial effects, including antioxidation, antisepsis, antihepatic fibrosis, neuro-protection, depressurization, immune regulation, and analgesic effects [17,18]. Despite the diverse functions of Ori that have attracted significant research attention in recent years, there is limited investigation into its potential role during *MTB* infection. Therefore, this study aims to evaluate the impact of Ori on mycobacterial infection-induced oxidative stress and its potential function as an antimycobacterial agent. Our findings suggest that Ori significantly inhibits *Mm* entry into lung epithelial cells and proliferation in zebrafish. Meanwhile, Ori reduces the inflammatory response and oxidative stress caused by *Mm* infection. These results enhance our understanding of the pharmacological function of Ori, highlighting its potential as a potent therapeutic candidate for treating or managing TB. Further, these results expand our understanding of the pharmacological function of Ori and suggest the ent-kaurane diterpenoid as a strong therapeutic candidate in the treatment or remission of TB.

## 2. Materials and Methods

### 2.1. Cell Culture and CCK8 Assay

RAW264.7 cells (mouse macrophages, BNCC354753) and A549 cells (human alveolar epithelial cells, BNCC337696), purchased from BeNa Culture Collection (Beijing, China), were cultured in Dulbecco’s modified Eagle’s medium (Gibco, Grand Island, NY, USA) supplement with 10% fetal bovine serum (Gibco, Grand Island, NY, USA) and RPMI1640 medium (Gibco, Grand Island, NY, USA) supplemented with 10% fetal bovine serum, respectively. The cells were maintained at 37 °C in a humidified chamber of 5% CO_2_.

A549 cells and RAW264.7 cells (1 × 10^4^/well) were seeded in 96-well plates and cultured overnight. The cells were pre-treated with varying concentrations of Ori for 24 h, followed by stimulation with CCK8 solution (1 mg/mL) for 30 min. Subsequently, dimethyl sulfoxide (DMSO) was added to each well (2 μL/well). Absorbance was measured at a wavelength of 450 nm using a microplate reader (Flex Station 3 microplate reader; Molecular Devices, Sunnyvale, CA, USA).

### 2.2. Mm Culture

The pTEC27 plasmid [19], which contains the td-Tomato protein, is a kind gift from Lalita Ramakrishnan (Department of Medicine, University of Cambridge, Cambridge, UK) and used to transform into *Mm* by electroporation. The *Mm* and *Wasabi-Mm* strains were obtained from Professor Chen Niu (School of Basic Medical Sciences, Fudan University, Shanghai, China) and cultured in 7H9 broth supplemented with 10% Middlebrook OADC Enrichment (Becton Dickinson and Company, NY, USA) and 0.05% Tween 80 (Solarbio, Beijing, China) at 30 °C.

### 2.3. Mm Infection and Colony-Forming Units (CFU) Assay

A549 cells were seeded in 24-well plates and cultured to approximately 80% confluency. The cells were then treated with Ori for 1 h, followed by infection with *Mm* for 4 h. After removing the cell culture, the cells were washed cold PBS five times (each time 5 min). Subsequently, a solution of 0.01% Triton X-100 (Solarbio, Beijing, China) was used to lyse the cells and release *Mm*. The lysed process was terminated by adding 800 μL of PBS to the solution, which was then collected and diluted tenfold before being coated on 7H10 agar plates and incubated at a temperature of 30 °C for 1–2 weeks.

### 2.4. Microinjection of Mm into Zebrafish

E3 media containing 5 mM NaCl, 0.17 mM KCl, 0.33 mM CaCl_2_, 0.33 mM MgSO_4_, and 10% methylene blue were used to culture the embryos of zebrafish. Td-Tomato-*Mm* or wasabi-*Mm* were microinjected into the caudal vein of embryos at 28–30 hpf, as previously described [19]. Briefly, well-developed embryos were selected after collection in E3 medium and addition of 0.003% Phenylthiourea (PTU) (12–18 h zebrafish). We chose well-developed embryos, removing the embryonic membrane and anaesthetizing the larvae with tricaine before injecting 100 CFU/mL *Mm* into zebrafish via caudal vein microinjection (28–32 h zebrafish). The zebrafish were then transferred to fresh E3 medium and divided into two groups. The group was treated or non-treated with 35 μM Ori for 7 days. The images were acquired using a Zeiss LSM 710 NLO Multiphoton microscope (Carl Zeiss, Jena, Germany). Four zebrafish were selected and placed in an EP tube, followed by the addition of 485 μL PBS and 15 μL kanamycin. The tube was then placed on a metal bath at 27 °C for 45 min, homogenized, and the resulting mixture was coated on 7H10 agar plates for culture 2 weeks.

### 2.5. RNA Isolation and qRT-PCR

Total RNA was extracted from cells using Trizol reagent (Invitrogen, Carlsbad, CA, USA) according to the manufacturer’s protocol. Total RNA (1 μg) was reverse transcribed into cDNA using the TransScript first-strand cDNA synthesis SuperMix (TransGen Biotech, Beijing, China). Real-time quantitative PCR was performed using SYBR^®^ Premix Ex Taq™ II (Tli RNase H Plus) (TaKaRa, Dalian, China) by ABI PRISM^®^7500 real-time PCR system (Applied Biosystems, Foster City, CA, USA). The primers used in this study were designed using Primer 5 and synthesized by Sangon Biotech Co., Ltd., (Shanghai, China). The primer sequences were as follow: TNF-α, F-5′-GCAACTGCTGCACGAAATC-3′, R-5′-CTGCTTGTCCTCTGCCCAC-3′; IL-6, F-5′-CCAGAAACCGCTATGAAGTTCC-3′, R-5′-GTTGGGAGTGGTATCCTCTGTGA-3′; IL-1β, F-5′-TGTGATGTTCCCATTAGAC-3′, R-5′-AATACCACTTGTTGGCTTA-3′; and β-actin, F-5′-AGTGTGACGTGGACATCCGCA-3′, R-5′-ATCCACATCTGCTGGAAGGTGGAC-3′.

### 2.6. Western Blot Analysis

Radio immunoprecipitation lysis buffer (RIPA, Solarbio, Beijing, China) supplement with 1% phenylmethylsulfonyl fluoride (PMSF) and phosphatase inhibitors (Solarbio, Beijing, China) were used to treat RAW264.7 cells at 4 °C for 30 min, then centrifuged at 12,000× *g* at 4 °C for 10 min before we collected the supernatant. The concentration of supernatant protein was quantified with a Pierce BCA protein Assay Kit (Thermo Fisher Scientific, Rockford, IL, USA). Equal proteins were separated by 10% SDS-PAGE gels through electrophoresis and then transferred to 0.45 μM PVDF membranes (Millipore, Billerica, MA, USA). After blocking with 5% non-fat milk (Solarbio, Beijing, China) in TBST, the membranes were incubated with primary antibody at 4 °C overnight. The primary antibodies against NRF2 p-AKT, AKT, iNOS (Cell Signaling Technology, Danvers, MA, USA), and HO-1, NQO-1, COX-2, p-GSK-3β, GSK3β, p-AMPK-α1, AMPK-α1, and β-actin (ABclonal, Wuhan, China) were employed. The dilution ratio of all primary antibodies is 1:2000. The membranes were washed four times with TBST (15 min each time). Next, the membranes were incubated with secondary goat anti-rabbit (1:10,000, ABclonal, Wuhan, China) and goat anti-mice (1:10,000, ABclonal, Wuhan, China) antibody for 1 h at room temperature. Membranes were visualized using enhanced chemiluminescence (ECL kit; Applygen Inst. Biotech, Beijing, China) by Amersham Imager600 (a gel imaging system form GE Co.). ImageJ software was used to numericize the western blot results.

### 2.7. Statistics

Statistical analysis was performed using SPSS 12.0 (SPSS Inc., Chicago, IL, USA). Groups comparisons were conducted by one-way analysis of variance (ANOVA) followed by the least significant difference test. * *p* <0.05 was considered significant, and ** *p* < 0.01 was considered markedly significant. Two-group comparisons were analyzed using Student’s *t* test.

## 3. Results

### 3.1. Ori Hinders Mm Entry into Lung Epithelial Cells

**Figure 2:**
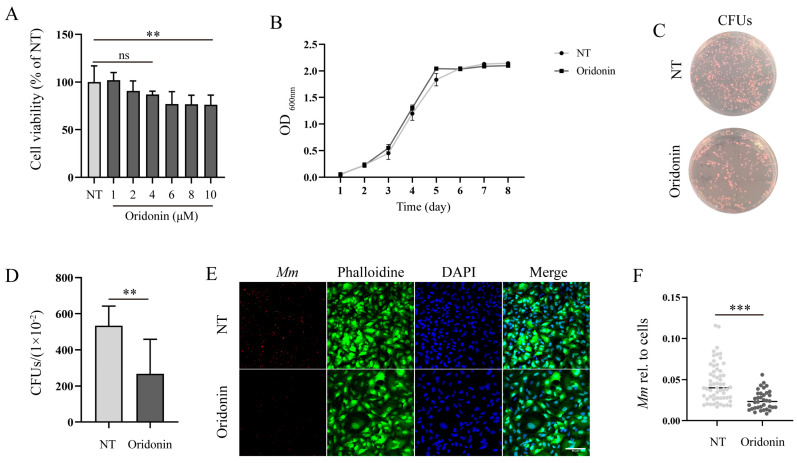
Ori inhibits *Mm* infection in A549 cells. (**A**) The cytotoxicity of Ori (1, 2, 4, 6, 8, and 10 μM) on A549 cells, “ns” representative no significance, (*n* = 6. (**B**) The growth performance of *Mm* was assessed after treatment with 2 μM Ori for 7 d. (**C**) Pretreatment of A549 cells with Ori for 1 h followed by infection with *Mm* for 6 h. We collected and disrupted the cells using Triton X-100. The lysate of A549 cells was diluted by one hundred times and subsequently coated on 7H10 agar plates. MOI is 10. (**D**) Quantification of CFUs results (*n* ≥ 6). (**E**) After treatment the A549 cells with 2 μM Ori, immunofluorescence analysis reveals *Mm* entry into A549 cells. The MOI of *Mm* is 10. Magnification shown is 20×, and the scale bar represents 50 μm. (**F**) Using Image J quantified *Mm* immunofluorescence intensity in images of *Mm* infected A549 cells and calculated the area of *Mm*/the area of the cell nucleus, each dot representing one image (*n* ≥ 36). The results shown are means ± SEM, ** *p* < 0.01, and *** *p* < 0.001.

In this study, we aim to investigate the impact of Ori inhibition on *Mm* infection in alveolar epithelial cells. First, we evaluated the effects of Ori on the viability of A549 cells and found that while does of 1, 2, and 4 μM had no effects on A549 cells viability, concentrations of 6, 8, and 10 μM significantly reduced cell viability. To ensure Ori has no effect on cell activity, 2 μM of Ori was used to treat cells. A 7-day incubation period with 2 μM of Ori had no significant effects on *Mm* growth performance (Figure 2B). Subsequently, the influence of Ori on *Mm* infection alveolar epithelial cells was evaluated. The CFU results demonstrated that Ori significantly impedes the entry of *Mm* into A549 cells (Figure 2C,D). To further validate these findings, we utilized laser scanning confocal microscopy to examine images of Mm-infected cells and digitized the images using Image J software. Our results showed that Ori markedly inhibits *Mm* entry into A549 cells (Figure 2E,F), suggesting its potential as an anti-infective agent against *Mm* infection.

### 3.2. Ori Inhibits the Pro-Inflammatory Response Induced by Mm Infection in RAW264.7 Cells

**Figure 3:**
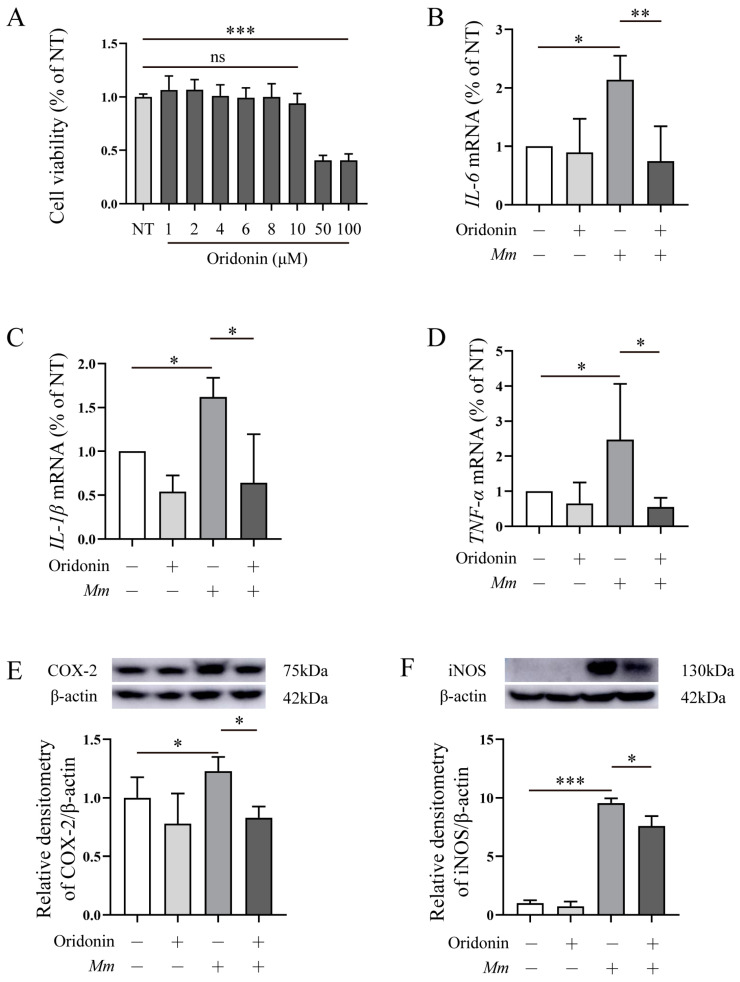
Ori attenuates *Mm* infection-induced inflammatory response in RAW264.7 cells. (**A**) The cytotoxicity of Ori (1, 2, 4, 6, 8, 10, 50, and 100 μM) on RAW264.7 cells, “ns” representative no significance, (*n* = 6). (**B**–**D**) RAW264.7 cells were pretreated using Ori for 1 h followed by infection with *Mm* for 6 h, the MOI of *Mm* is 10. Next, qRT-PCR was employed to analyze the gene expression of pro-inflammatory cytokines *IL-6*, *IL-1β*, and *TNF-α* (*n* = 3). (**E**–**F**) RAW246.7 cells were treated with Ori for 1 h followed by infecting with *Mm* for 12 h, and western blot was performed to assess the expression of iNOS and COX-2. The MOI of *Mm* is 10 (*n* = 3). “+” representative adding Ori or *Mm*, “–” representative non-treatment. The results shown are means ± SEM, * *p* < 0.05, ** *p* < 0.01, and *** *p* < 0.001.

Next, we conducted experiments on *Mm*-infected RAW264.7 cells to investigate the anti/pro-inflammatory function of Ori. Firstly, we assessed the impact of Ori on the viability of RAW264.7 cells using CCK8. The results revealed that at doses of 1, 2, 4, 6, 8, and 10 μM, Ori had no significant effects on cell viability; however, at concentrations of 50 and 100 μM, Ori markedly decreased RAW264.7 cells viability (Figure 3A). Hence, 8 μM Ori was used in the subsequent experiments. RAW264.7 cells were pretreated with Ori for 1 h followed by infecting with *Mm* for 6 h. The qRT-PCR results showed that *Mm* infection upregulates the expression of pro-inflammatory cytokines *IL-6*, *IL-1β*, and *TNF-α*, and pretreatment with Ori effectively inhibits their expression (Figure 3B–D). Additionally, we assessed the expression of iNOS, and COX-2 in *Mm*-infected RAW264.7 cells following pretreatment with Ori. The Western blot results revealed that Ori demonstrated that Ori effectively inhibited *Mm* infection-induced upregulation of iNOS and COX-2 (Figure 3E,F). Collectively, these findings suggest that Ori exerts anti-inflammatory effects in *Mm*-infected RAW264.7 cells.

### 3.3. Ori Inhibits the Proliferation of Mm in Zebrafish and Alleviates Oxidative Stress in Mm-Infected Zebrafish

**Figure 4:**
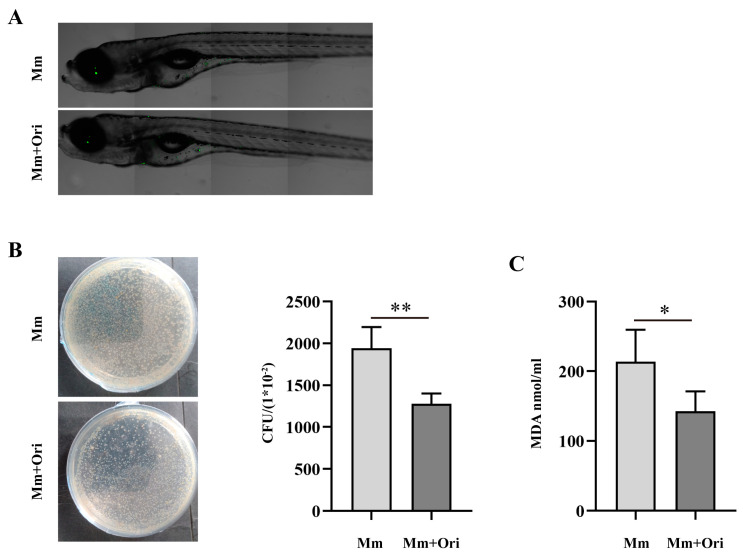
Ori inhibits *Mm* proliferation in zebrafish, and *Mm* infection induces oxidative stress. (**A**) Ori was added into the E3 medium of zebrafish to a final concentration of 35 μM. Mm was injected into the zebrafish by caudal vein microinjection 7 days later, scanning the zebrafish with laser scanning confocal microscope (*n* ≥ 5). (**B**) Supplement with Ori in E3 medium of zebrafish, and *Mm* was injected into the zebrafish by caudal vein microinjection and cultured for 7 days. Four zebrafish were selected and transferred into the EP tube which contained 485 μL PBS and 15 μL kanamycin. The tube was then placed on a metal bath at 27 °C for 45 min and homogenized, and the resulting mixture was coated on 7H10 agar plates for 2 weeks’ culture. (**C**) The impact of Ori on *Mm* infection-induced MDA in zebrafish was evaluated (*n* ≥ 20). The results shown are means ± SEM, * *p* < 0.05, ** *p* < 0.01.

In this study, we identified that Ori inhibits *Mm* infection into cells. Next, we performed the test on the effects of Ori on *Mm* infection zebrafish. First, we evaluated the different concentration of Ori on survival of zebrafish. Our findings indicated that 100 μM and 200 μM Ori significantly reduce the survival rate of zebrafish (Figure S1A). Further investigation revealed that the presence of 20–40 μM Ori did not exert any discernible impact on the survival, growth, and development of zebrafish (Figure S1A,B). Consequently, a concentration of 35 μM of Ori was selected for subsequent experiments. Ori was added into the E3 medium of zebrafish to a final concentration of 35 μM; then, *Mm* was injected into the zebrafish by caudal vein microinjection. Seven days later, we proceeded to evaluate, either by observation or measurement, the bacilli load of *Mm* in zebrafish. The fluorescence scanning results demonstrated that Ori supplementation reduces the bacilli load of *Mm* in zebrafish (Figure 4A). Furthermore, CUF results also indicated that Ori supplementation inhibits the proliferation of *Mm* in zebrafish (Figure 4B). In addition, our findings revealed that Ori decreased the concentration of MDA in *Mm*-infected zebrafish (Figure 4C), suggesting its potential to mitigate oxidative stress in *Mm*-infected zebrafish.

### 3.4. The Effects of Ori on NRF2/HO-1/NQO-1, and AKT/GSK-3β/AMPK-α1 Signaling Pathways

**Figure 5:**
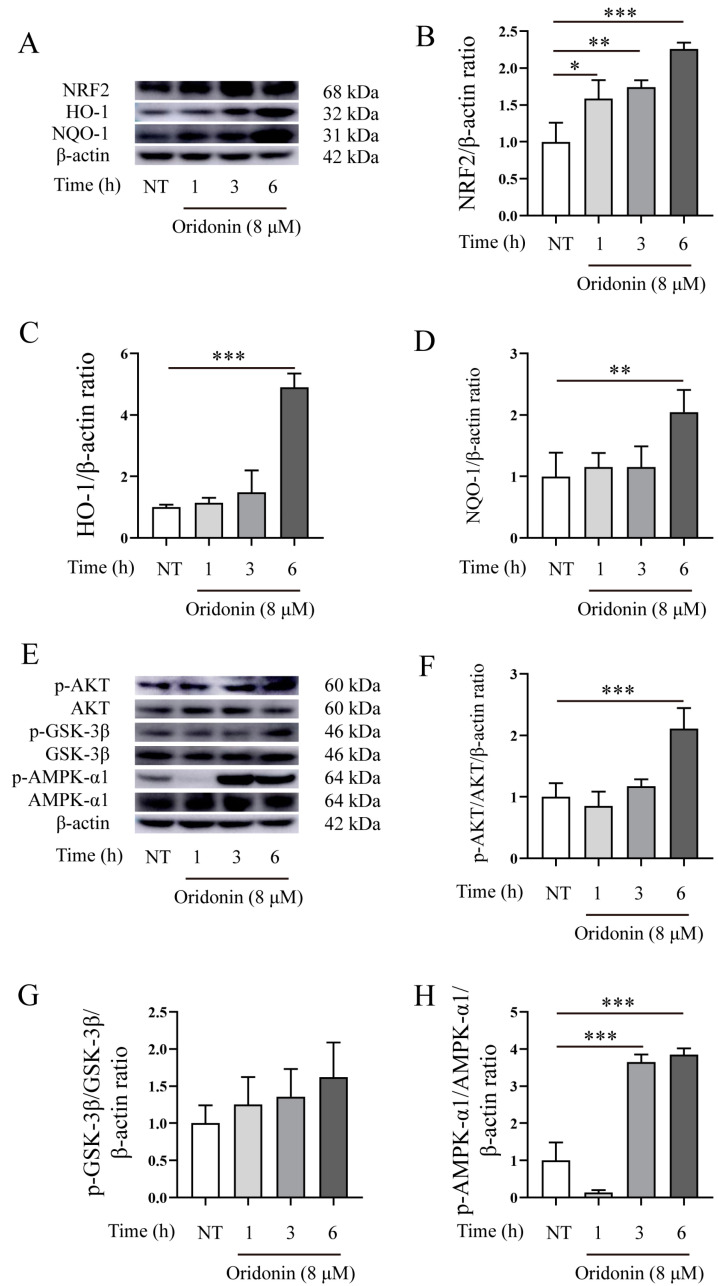
Ori upregulated the expression of NRF2/HO-1/NQO-1 and increased the phosphorylation of AKT/AMPKα/GSK-3β signaling pathway. (**A**–**D**) RAW246.7 cells were treated using Ori for 1, 3, and 6 h, and total protein was collected for western blot analysis of the expression of NRF2/HO-1/NQO-1 (*n* ≥ 3). (**E**–**H**) RAW246.7 cells were treated using Ori for 1, 3, and 6 h, and total protein was collected for Western blot analysis the phosphorylation of AKT/AMPKα/GSK-3β signaling pathway (*n* ≥ 3). The results shown are means ± SEM, * *p* < 0.05, ** *p* < 0.01, and *** *p* < 0.001.

Nrf2 is the most important transcription factor against oxidative stress and plays a crucial role in antioxidants and in the relief of inflammation [20]. Therefore, we evaluated the effects of Ori on NRF2, as well as its downstream HO-1 and NQO-1. Our findings demonstrated that treatment with Ori significantly enhance the expression of NRF2, HO-1, and NQO-1 in RAW264.7 cells (Figure 5A–D), suggesting that Ori may exert antioxidative effects. The AMP-activated protein kinase (AMPK)-mediated inactivation of glycogen synthase kinase 3β (GSK3β) promoted the nuclear accumulation of Nrf2 [21]. Furthermore, AMPK promotes the phosphorylation of PI3K/Akt and GSK-3β signaling. Therefore, we conducted the assays on RAW264.7 cells treatment with Ori to evaluate its antioxidative effects. The western blot results demonstrated that treatment with Ori significantly increased the phosphorylation of the AKT/AMPKα/GSK-3β signaling pathway (Figure 5E–H). Collectively, these findings suggest that Ori exerts an antioxidative effect in RAW264.7 cells.

## 4. Discussion

Tuberculosis (TB) is a highly communicable disease caused by *MTB*. According to the Global Tuberculosis Report, an estimated 10.6 million people fell ill with TB in 2021, an increase of 4.5% from 2020. Approximately 90% of the new TB cases occur in adults, and males are more commonly affected than females. Prior to the COVID-19 pandemic, TB was the leading cause of death from a single infectious agent, ranking above HIV/AIDS with its toll of 1.6 million deaths in 2021 alone [22]. Undoubtedly, TB remains a global public health concern that poses a threat to human well-being [2,3], and searching for new anti-TB drugs remains urgent. zebrafish is a natural host for *Mm*, a close genetic relative of *Mtb* that causes TB [23]. Similar to *MTB*, *Mm* replicates within zebrafish macrophages and induces chronic granulomatous infection through shared virulence determinants [24]. The use of Mm-infected zebrafish larvae as a model system recapitulates key aspects of TB pathogenesis and drug treatment, making it an ideal platform for identifying novel antibacterial agents and entirely new classes of drugs against *MTB* infection. Therefore, in this study, we utilized *Mm*-infected zebrafish as an in vivo model to investigate the anti-tuberculosis properties of Ori.

Ori, a bioactive ent-kaurane diterpenoid compound derived from the leaves of *Rabdosia rubescens*, has been identified as exhibiting antibacterial properties. Previous studies showed that Ori inhibits the release of cytokines IL-2, IL-4, IL-6, IL-10, and TNF-α, and maintains Th1/Th2 balance in the spleen of *Salmonella pullorum* infected Arbor Acres broilers [25,26]. Supplement with Ori significantly enhanced the immune response and maintained proper intestinal health in Arbor Acres broilers infected with *Salmonella pullorum* [27]. The derivative of Ori show great potential properties as an agent against *Bacillus subtilis*, being nearly threefold stronger than positive control chloromycetin [28]. The enmein-type derivatives of Ori exhibit potent activity against *Mycobacterium phlei*, albeit with reduced efficacy against *Mycobacterium smegmatis* and *Mm* [29]. Collectively, these findings suggest that Ori possesses antibacterial properties; however, its effects on mycobacteria remain understudied.

In this study, we evaluated the impact of Ori on *Mm* infection, *Mm*-infected cells, and zebrafish. Our findings showed that Ori does not significantly affect *Mm* growth performance (Figure 2B), which may be attributed to its low concentration (this concentration of Ori depends on the effects of Ori on cell viability). However, the CFUs and laser confocal scanning results demonstrated that 2 μM Ori significantly reduces the infection of *Mm* to lung epithelial cells (Figure 2C–F), suggesting a potential antibacilli infection property for Ori. The inflammatory response to *MTB* is a dynamic process that potentially involves the continuous migration of cells into and out of the granuloma, and appropriate regulation of this response is crucial for controlling *MTB*. However, the excessive inflammatory response and overexpression of inflammatory cytokines may lead to cell death and severe inflammatory damage. Therefore, maintaining pro-inflammatory cytokines at an appropriate level may provide help in the control of TB. Ori has been shown to ameliorate acute kidney injury by inhibiting macrophages-mediated inflammation [30], and suppressing the expression of cytokines IL-2, IL-4, IL-6, IL-10, and TNF-α [25], suggesting that Ori had a potent anti-inflammatory response activity. In this study, Ori significantly decreased the expression of pro-inflammatory cytokines TNF-α, IL-6, and IL-1β, and pro-inflammatory enzymes COX-2 and Inos (Figure 3A–F). These findings indicated that Ori inhibits the *Mm* infection-induced inflammatory response in macrophages. To investigate the effects of Ori on *Mm*-infected zebrafish, we first evaluated its impact on the growth and development of zebrafish. Previous studies indicated that concentrations of 100, 200, and 400 mg/L of Ori have adverse effects on the early stages of zebrafish development [31]. Our findings demonstrated that 20–40 μM (7.29–14.58 mg/L) of Ori has no effects on the survival, growth, and development of early stages zebrafish (Figure S1A,B). Subsequently, we administered Ori into E3 medium of zebrafish, and *Mm* were injected into the zebrafish by caudal vein microinjection. The CFU and laser confocal scanning results showed that 35 μM Ori significantly reduced the bacilli burden in zebrafish (Figure 4A,B), suggesting that Ori inhibited the proliferation of *Mm* in zebrafish. Although the specific mechanism remains unknown, these findings provide compelling evidence for further development of Ori as an anti-TB drug. Supplementation with Ori reduced the levels of SOD2, CAT, MDA, ROS levels, and IL-1β, TNF-α in hind limb muscle tissue of ischemia–reperfusion injury mice [32], suggesting that Ori plays a protective role against oxidative stress and the inflammatory response. Our study revealed that supplementation with Ori significantly reduced the content of MDA in *Mm*-infected zebrafish (Figure 4C), suggesting that Ori may exert inhibitory effects on oxidative stress in *Mm*-infected zebrafish. NRF2 is the most important transcription factor against oxidative stress, and it plays a crucial role in antioxidants and anti-inflammatory processes [20]. AMPK-mediated inactivation of GSK-3β promotes the nuclear accumulation of NRF2 [21]. Additionally, AMPK promotes the phosphorylation of PI3K/AKT and the GSK-3β signaling pathway. In this study, supplementation with Ori increased the expression of NRF2/HO-1/NQO-1 and the phosphorylation of AKT/AMPK-α1/GSK-3β signaling pathway.

## 5. Conclusions

In summary, our findings demonstrated that Ori provides a protective role during *Mm* infection. Specifically, Ori exhibits inhibitory effects on *Mm* proliferation in zebrafish, which provides strong evidence for its potential use as an anti-TB drug. Moreover, the inhibitory effects of Ori on *Mm* infection-induced oxidative stress may be related to the upregulation of NRF2/HO-1/NQO-1 protein and activation of the AKT/AMPK-α1/GSK-3β signaling pathway.

## Data Availability

All data are contained within the article.

## Supplementary Materials

The following supporting information can be downloaded at: https://www.mdpi.com/article/10.3390/pathogens12060799/s1, Figure S1: The effects of Ori on the survival, growth and development of zebrafish.

## References

[1] Suliman S., Pelzer P.T., Shaku M., Rozot V., Mendelsohn S.C. (2021). Meeting report: Virtual Global Forum on Tuberculosis Vaccines, 20–22 April 2021. Vaccine.

[2] Dutta N.K., Klinkenberg L.G., Vazquez M.J., Segura-Carro D., Colmenarejo G., Ramon F., Rodriguez-Miquel B., Mata-Cantero L., Porras-De Francisco E., Chuang Y.M. (2019). Inhibiting the stringent response blocks Mycobacterium tuberculosis entry into quiescence and reduces persistence. Sci. Adv..

[3] Nathavitharana R.R., Friedland J.S. (2015). A tale of two global emergencies: Tuberculosis control efforts can learn from the Ebola outbreak. Eur. Respir. J..

[4] World Health Organization (2018). Latent Tuberculosis Infection: Updated and Consolidated Guidelines for Programmatic Management.

[5] Emery J.C., Richards A.S., Dale K.D., McQuaid C.F., White R.G., Denholm J.T., Houben R. (2021). Self-clearance of Mycobacterium tuberculosis infection: Implications for lifetime risk and population at-risk of tuberculosis disease. Proc. Biol. Sci..

[6] Behr M.A., Edelstein P.H., Ramakrishnan L. (2019). Is Mycobacterium tuberculosis infection life long?. BMJ.

[7] Lagman M., Ly J., Saing T., Kaur Singh M., Vera Tudela E., Morris D., Chi P.T., Ochoa C., Sathananthan A., Venketaraman V. (2015). Investigating the causes for decreased levels of glutathione in individuals with type II diabetes. PLoS ONE.

[8] Zumla A., George A., Sharma V., Herbert R.H., Ilton B.M.O., Oxley A., Oliver M. (2015). The WHO 2014 global tuberculosis report—Further to go. Lancet. Glob. Health.

[9] Ramappa V., Aithal G.P. (2013). Hepatotoxicity Related to Anti-tuberculosis Drugs: Mechanisms and Management. J. Clin. Exp. Hepatol..

[10] Singh P., Subbian S. (2018). Harnessing the mTOR Pathway for Tuberculosis Treatment. Front. Microbiol..

[11] Kuo L.M., Kuo C.Y., Lin C.Y., Hung M.F., Shen J.J., Hwang T.L. (2014). Intracellular glutathione depletion by oridonin leads to apoptosis in hepatic stellate cells. Molecules.

[12] Sarwar M.S., Xia Y.X., Liang Z.M., Tsang S.W., Zhang H.J. (2020). Mechanistic Pathways and Molecular Targets of Plant-Derived Anticancer ent-Kaurane Diterpenes. Biomolecules.

[13] Chen K., Ye J., Qi L., Liao Y., Li R., Song S., Zhou C., Feng R., Zhai W. (2019). Oridonin inhibits hypoxia-induced epithelial-mesenchymal transition and cell migration by the hypoxia-inducible factor-1alpha/matrix metallopeptidase-9 signal pathway in gallbladder cancer. Anti-Cancer Drugs.

[14] Li J., Wu Y., Wang D., Zou L., Fu C., Zhang J., Leung G.P. (2019). Oridonin synergistically enhances the anti-tumor efficacy of doxorubicin against aggressive breast cancer via pro-apoptotic and anti-angiogenic effects. Pharmacol. Res..

[15] Zhang Y., Wang S., Dai M., Nai J., Zhu L., Sheng H. (2020). Solubility and Bioavailability Enhancement of Oridonin: A Review. Molecules.

[16] Li J., Bao L., Zha D., Zhang L., Gao P., Zhang J., Wu X. (2018). Oridonin protects against the inflammatory response in diabetic nephropathy by inhibiting the TLR4/p38-MAPK and TLR4/NF-kappaB signaling pathways. Int. Immunopharmacol..

[17] Xu J., Wold E.A., Ding Y., Shen Q., Zhou J. (2018). Therapeutic Potential of Oridonin and Its Analogs: From Anticancer and Antiinflammation to Neuroprotection. Molecules.

[18] Li X., Zhang C.T., Ma W., Xie X., Huang Q. (2021). Oridonin: A Review of Its Pharmacology, Pharmacokinetics and Toxicity. Front. Pharmacol..

[19] Takaki K., Davis J.M., Winglee K., Ramakrishnan L. (2013). Evaluation of the pathogenesis and treatment of Mycobacterium marinum infection in zebrafish. Nat. Protoc..

[20] Kobayashi E., Suzuki T., Yamamoto M. (2013). Roles nrf2 plays in myeloid cells and related disorders. Oxidative Med. Cell. Longev..

[21] Rizvi F., Mathur A., Kakkar P. (2015). Morin mitigates acetaminophen-induced liver injury by potentiating Nrf2 regulated survival mechanism through molecular intervention in PHLPP2-Akt-Gsk3beta axis. Apoptosis Int. J. Program. Cell Death.

[22] Bagcchi S. (2023). WHO’s Global Tuberculosis Report 2022. Lancet. Microbe.

[23] Stinear T.P., Seemann T., Harrison P.F., Jenkin G.A., Davies J.K., Johnson P.D., Abdellah Z., Arrowsmith C., Chillingworth T., Churcher C. (2008). Insights from the complete genome sequence of Mycobacterium marinum on the evolution of Mycobacterium tuberculosis. Genome Res..

[24] Cosma C.L., Klein K., Kim R., Beery D., Ramakrishnan L. (2006). Mycobacterium marinum Erp is a virulence determinant required for cell wall integrity and intracellular survival. Infect. Immun..

[25] Wu Q.J., Zheng X.C., Wang T., Zhang T.Y. (2018). Effects of oridonin on immune cells, Th1/Th2 balance and the expression of BLys in the spleens of broiler chickens challenged with Salmonella pullorum. Res. Vet. Sci..

[26] Wu Q.J., Zheng X.C., Wang T., Zhang T.Y. (2018). Effects of dietary supplementation with oridonin on the growth performance, relative organ weight, lymphocyte proliferation, and cytokine concentration in broiler chickens. BMC Vet. Res..

[27] Wu Q.J., Zheng X.C., Wang T., Zhang T.Y. (2018). Effect of dietary oridonin supplementation on growth performance, gut health, and immune response of broilers infected with Salmonella pullorum. Ir. Vet. J..

[28] Li D., Han T., Xu S., Zhou T., Tian K., Hu X., Cheng K., Li Z., Hua H., Xu J. (2016). Antitumor and Antibacterial Derivatives of Oridonin: A Main Composition of Dong-Ling-Cao. Molecules.

[29] Xu S., Pei L., Li D., Yao H., Cai H., Yao H., Wu X., Xu J. (2014). Synthesis and antimycobacterial evaluation of natural oridonin and its enmein-type derivatives. Fitoterapia.

[30] Tan R.Z., Yan Y., Yu Y., Diao H., Zhong X., Lin X., Liao Y.Y., Wang L. (2021). Renoprotective Effect of Oridonin in a Mouse Model of Acute Kidney Injury via Suppression of Macrophage Involved Inflammation. Biol. Pharm. Bull..

[31] Tian L., Sheng D., Li Q., Guo C., Zhu G. (2019). Preliminary safety assessment of oridonin in zebrafish. Pharm. Biol..

[32] Zhao X., Liu Y., Wang L., Yan C., Liu H., Zhang W., Zhao H., Cheng C., Chen Z., Xu T. (2022). Oridonin attenuates hind limb ischemia-reperfusion injury by modulating Nrf2-mediated oxidative stress and NLRP3-mediated inflammation. J. Ethnopharmacol..

