# Supplementary material for: Oridonin Inhibits Mycobacterium marinum Infection-Induced Oxidative Stress In Vitro and In Vivo
Source: Pathogens. 2023 Jun 3;12(6):799. doi: 10.3390/pathogens12060799 (PMC10301349; doi:10.3390/pathogens12060799)
Supplement: Supplementary file 1 [file pathogens-12-00799-s001.zip › pathogens-2353210-supplementary.pdf]

Supplementary materials

Figure S1

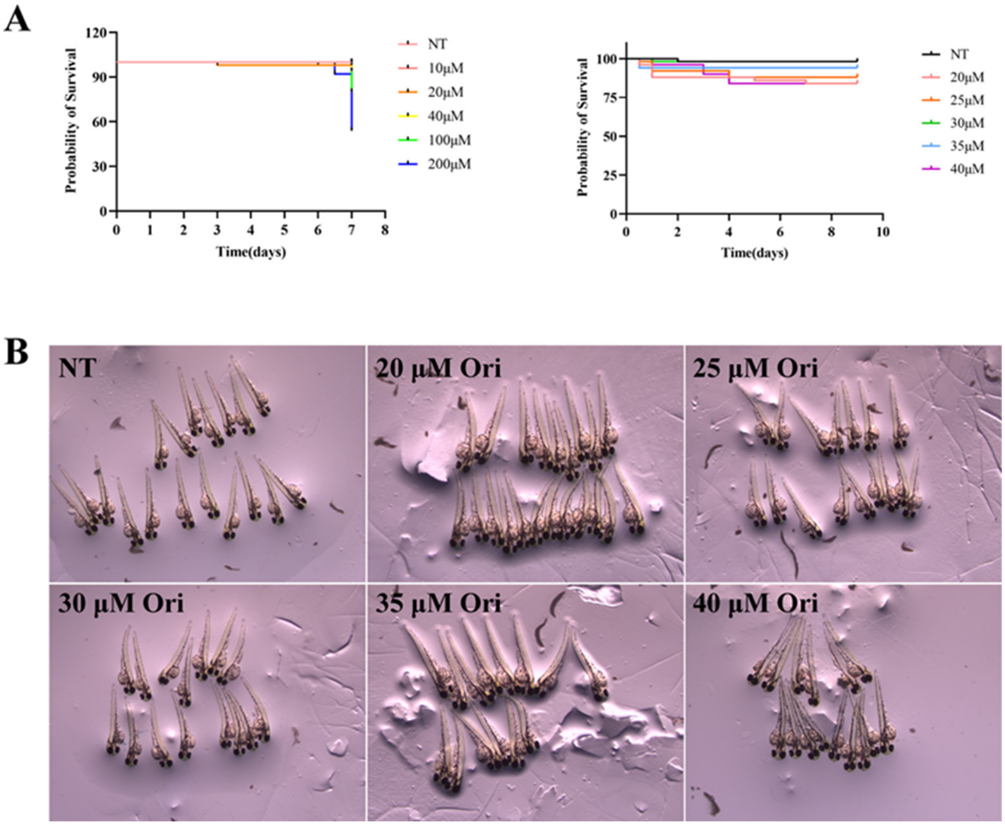

Figure S1. The effects of Ori on the survival, growth, and development of zebrafish. (**A**) The effects of varying concentrations of Ori on the survival of zebrafish (n≥50). (**B**) The effects of different concentrations of Ori on the growth and development of zebrafish (n≥50).
